# Functions of Peptidoglycan Recognition Proteins (Pglyrps) at the Ocular Surface: Bacterial Keratitis in Gene-Targeted Mice Deficient in *Pglyrp*-2, -3 and -4

**DOI:** 10.1371/journal.pone.0137129

**Published:** 2015-09-02

**Authors:** Ranjita N. Gowda, Rachel Redfern, Jihane Frikeche, Sudarshan Pinglay, James William Foster, Carolina Lema, Leslie Cope, Shukti Chakravarti

**Affiliations:** 1 Department of Medicine, Johns Hopkins University School of Medicine, Baltimore, MD, United States of America; 2 The Ocular Surface Institute, College of Optometry, University of Houston, Houston, TX, United States of America; 3 Department of Cell Biology, Johns Hopkins University School of Medicine, Baltimore, MD, United States of America; 4 Department of Ophthalmology, Johns Hopkins University School of Medicine, Baltimore, MD, United States of America; 5 Department of Oncology, Johns Hopkins University School of Medicine, Baltimore, MD, United States of America; University of Illinois at Chicago, UNITED STATES

## Abstract

**Purpose:**

Functions of antimicrobial peptidoglycan recognition proteins (Pglyrp1-4) at the ocular surface are poorly understood. Earlier, we reported an antibacterial role for *Pglyrp*-1 in *Pseudomonas aeruginosa* keratitis. Here we investigated functions of three other related genes *Pglyrp*-2, -3 and -4 in a mouse model of *P*. *aeruginosa* keratitis.

**Methods:**

Wild type (WT) and each of the *Pglyrp*-null genotypes were challenged with *P*. *aeruginosa* keratitis. The eyes were scored in a blinded manner 24 and 48h post infection. Viable bacterial counts and inflammatory factors (IL-12, TNF-α, IFN-γ, CCL2, IL-6 and IL-10) were measured in whole eye homogenates using cytometric bead arrays. Expressions of *Pglyrp-1-4*, mouse beta defensins (*mBD)-2*,*-3*, cathelicidin-related antimicrobial peptide (CRAMP) were determined by qRTPCR in total RNA extracts of uninfected and infected eyes of WT and each of the *Pglyrp*-null mouse types.

**Results:**

The *Pglyrp-2* ^*-/-*^ mice showed reduced disease and lower induction of pro-inflammatory TNF-α (*p* = 0.02) than WT or the other *Pglyrp* null mice. Viable bacterial yield was significantly lower in the *Pglyrp*-2^-/-^ (*p* = 0.0007) and the *Pglyrp*-4^-/-^ (*p* = 0.098) mice. With regards to expression of these antimicrobial genes, *Pglyrp*-2 expression was induced after infection in WT mice. *Pglyrp*-3 expression was low before and after infection in WT mice, while *Pglyrp*-4 expression was slightly elevated after infection in WT, *Pglyrp*-2 and -3 null mice. *Pglyrp*-1 expression was slightly elevated after infection in all genotypes without statistical significance. Transcripts for antimicrobial peptides mBD2, mBD3 and CRAMP were elevated in infected *Pglyrp-2* ^-/-^ males without statistical significance.

**Conclusions:**

Efficient resolution of keratitis in the *Pglyrp-2* ^*-/-*^ mice may be due to a reduced pro-inflammatory microenvironment and synergistic antibacterial activities of defensins, CRAMP and Pglyrp-1. Therefore, in ocular infections the pro-inflammatory functions of *Pglyrp*-2 must be regulated to benefit the host.

## Introduction

The peptidoglycan recognition proteins (Pglyrps) are conserved from insects to humans [1]. In insects this is a family of 19 proteins while in mammals there are only four genes that encode four secreted proteins [2, 3]. Human PGLYRP-1 is the smallest (196 aa), while PGLYRP-2 (576 aa), -3 (341 aa) and -4 (373 aa) are larger. PGLYRP-3 and -4 can form homodimers and heterodimers. All four mammalian members are antibacterial, have at least one Pglyrp domain in the C-terminal that shares homologies with bacterial type 2 amidases. In addition, all recognize gram-positive bacterial peptidoglycans, [4] but can also interact with gram-negative bacterial lipopolysaccharides and fungal cell wall components [3]. Mammalian Pglyrp-2 is the only remaining member with an n-acetylmuramoyl-l-alanine amidase activity that hydrolyzes peptidoglycans. Mice deficient in *Pglyrp*-1 have normal survival and body weight, but show increased susceptibility to intraperitoneal injections of *Bacillus subtilis* [5]. Pglyrp-1 is a major component of polymorphonuclear neutrophil (PMN) and eosinophil granules and *Pglyrp*-1^-/-^ mice PMN show normal bacterial uptake but poor bacterial clearance [5]. The Pglyrps also interact with cell surface pathogen recognition receptors such as the toll-like receptors and cytoplasmic Nod 1 [6, 7], thereby regulating the microbial population and the host inflammatory milieu. In a gene expression study of the cornea compared to tendon and the lens, we detected increased expression of *Pglyrp-1* in the cornea [8]. In a follow-up study we found constitutively high expression of *Pglyrp-1* in the superficial cells of the corneal epithelium in mouse and human corneas, and *Pglyrp-1* ^-/-^ mice challenged with *P*. *aeruginosa* keratitis showed poor bacterial clearance and resolution of keratitis [9]. These findings suggested a protective role for Pglyrp-1 at the ocular surface while functions of the other members, Pglyrp-2-4, at the ocular surface are largely unknown. *Pglyrp*-2 is expressed at high levels in the liver and the protein is secreted into the blood; in cultured epidermal and corneal keratinocytes, it was inducible by peptidoglycans [10, 11] Pglyrp-3 and -4 are widely present in various mucosal surfaces and in cultured corneal epithelial cells [7]. To investigate functions of Pglyrp-2-4 in ocular defense mechanisms, we investigated the response of gene-targeted mice deficient in *Pglyrp*-2, -3 and -4 to ocular infections with *P*. *aeruginosa*. Our study shows that disease severity is reduced with improved bacterial clearance in *Pglyrp-*2^-/-^ mice that may be due to compensatory over expression of defensins (*mBD2* and *3*), cathelicidin-related antimicrobial peptides (*Cnlp*) and *Pglyrp*-1.

## Materials and Methods

### Mice

Dr. Roman Dziarski (Department of Microbiology & Immunology, Indiana University School of Medicine, Indiana, USA) kindly provided mice deficient in *Pglyrp-2*, *Pglyrp-3* and *Pglyrp-4* genes in the BALB/c background. Wild-type BALB/c (WT) mice were purchased from Charles River (Charles River Laboratories, Wilmington, MA) and a colony was maintained in our facility for this study. Mice were housed in a specific pathogen-free facility of the Johns Hopkins University. The institutional Animal Care and Use Committee (IACUC) of the Johns Hopkins University specifically approved this study. Eight-ten week old male and female mice were used for these experiments. All experiments using mice were conducted in accordance with the ARVO Statement for the use of Animals in Ophthalmic and Vision Research.

### *Pseudomonas aeruginosa* Keratitis

The *P*.*aeruginosa* ATCC 19660 strain grown on cetrimide agar plates and ~ 0.5 x 10^8^ CFU/ml was used for corneal infections [12]. Mice were anesthetized with intraperitoneal injections of avertin (2,2,2-Tribromoethanol) and an inoculum of 5μl of the bacterial suspension was applied with a 26 gauge needle on one scarified cornea per animal as described previously [13]. The mice were examined in a blinded manner under a dissecting microscope (SMZ 1500; Nikon, Tokyo, Japan) fitted with a digital camera (DXM 1200; Nikon), and given a disease score based on a published scoring matrix [14]: 1- faint partial opacity, 2- dense opacity covering the pupil, 3- dense opacity over the entire eye, 4- corneal perforation and or phthisis bulbi. Clinical scores were obtained on all animals, while subsets were used for bacterial yield, cytokine analyses or histology. We used 5 different strains of mice with 4–5 independent experiments per strain, using 6–8 animals per experiment. This required extensive breeding, over a long period of time to generate a large number of animals to obtain age and gender matched mice for each experiment, and the experiments were carried out in batches over a period ~6 months. We included a set of WT control animals with each days experiment. Infected and un-infected eyes were photographed and selected representative eyes are shown. Viable bacterial yield was calculated by plating serial dilutions of whole eye homogenates in PBS from mice euthanized 2 days after infection.

### Quantitative Reverse Transcriptase Polymerase Chain Reaction (qRTPCR)

Total RNA was extracted from 6 animals per genotype from uninfected and 48 hour infected whole eyes in 500 μl of TRIzol (Invitrogen Corp., Carlsbad, CA) and first-strand cDNA was synthesized from 100ng of total RNA with reverse transcriptase (Superscript II; Invitrogen Corp.) and oligo (dT) primers and stored at −20°C until use. Real time qRTPCR was carried out using SYBR Green PCR master mix (Applied Biosystems) and the sequence detection system ABI PRISM 7900HT (Applied Biosystems, Foster City, CA). All primers used are shown in Table 1. The following PCR conditions were used for *Pglyrp-1-4*. The ramp rate was 1.6°C/sec; hold stage 1 was 50°C for 2 minutes and 95°C for 5 minutes; PCR stage at 95°C for 15 sec, 60°C for 30 sec, 72°C for 1 min; hold Stage 2 was at 72°C for 5 min; melt curve stage was at 95°C for 15 sec, 60°C for 1 min, 95°C for 15 sec. Expression relative to *Gapdh* (2^-ΔΔCT^) was calculated where ΔΔCT = (Ct _gene_-Ct_gapdh_) _infected eye_−(Ct _gene_-Ct_gapdh_) _uninfected eye_.

**Table 1:** Primers used in qRTPCR experiments.

Gene	Forward primer (5'-3')	Reverse primer (5'-3')
*Gapdh*	CCCTGGCCAAGGTCATCC	TGATGGCATGGACTGTGGTC
*Pglyrp1*	GCAATGTGCAGCATTACCAC	CTGTGTGGTCACCCTTGATG
*Pglyrp2*	TAGGCTCCGACGGCTATCTG	GTTCAGCGCAGCTTCGTT
*Pglyrp3*	GGCCTGTGGCAACCCAACCA	AGGGTACGGGCAGGCTCAGT
*Pglyrp4*	ACATCCAGCCATTGCTTGCGAA	CCGTGGAACTATGTGGGGACAAGC
*Hprt1*	CAGACTGAAGAGCTACTGTAATGA	CTTTCCAGTTAAAGTTGAGAGATCA
*Cramp*	TGAAGGCACATTGCTCAGGT	CCCAAGTCTGTGAGGTTCCG
*Mdb2*	CGAAAATCAGGGCTCAGTGC	TTCCCAGGTATCTTGCCAGC
*Mdb3*	CAGGCCCTTGGGCAAGTC	AGCTAAGTGGATTGAGTGCCT

For the three antimicrobial peptides, mouse beta-defensin 2 (mBD2), mouse beta-defensin 3 (mBD3) and cathelicidin-related antimicrobial peptide *Cnlp*-encoded CRAMP, 250 ng of total RNA was used to generate cDNA at 50°C for 60 min using iScript™ Reverse transcription Supermix (BIO-RAD). Samples containing no reverse transcriptase or water in place of RNA (no template control) served as negative controls. PCR amplification of cDNA was performed with SsoAdvanced™SYBR Green Supermix (BIO-RAD, Hercules, CA) using PrimePCR™SYBR Green specific primers for mBD2, mBD3, CRAMP, and HPRT (see Table 1). The following PCR conditions were used 95°C for 2 minutes (activation), and 40 cycles of 95°C for 5 seconds and 60°C for 30 seconds (denaturation, annealing/extension) using a CFX96™ Real-Time System (BIO-RAD). For each gene, the samples from six animals were processed in triplicate and relative fold change (2^-ΔΔCT^) was calculated with respect to *Hprt1*.

### H&E staining section

Eyes were enucleated and placed in 10% formalin/PBS, 5-mm corneal sections were prepared, and H & E staining was performed by the pathology core facility at the Johns Hopkins Medical Institutions (Reference Histology Laboratory).

### Cytometric Bead Array (CBA)

Cytokines IL-6, IL-10, MCP-1, IFN-γ, TNF, and IL-12p70 were measured using the Mouse Inflammation Kit BD™ Cytometric Bead Array (CBA) and the BD FACS Calibur (dual laser). The samples for the cytokines assays were prepared according to the manufacturer’s instructions and the flow cytometry results were analyzed using the FCAP Array^TM^ software.

### Statistical Analyses

In each keratitis experiment, all animals were given a clinical score, while subsets of animals were used for bacterial yield (approximately 5–9 mice per genotype), cytokine bead array assay (3 per genotype), qRTPCR (6 per genotype) and histology (3–5 animals per genotype). In each keratitis experiment either two or three genotypes (WT and one or two other null genotype) were tested at a time, and each genotype (n = 6–8 animals per genotype) was tested 5–6 times. All animals within an experiment were of one gender. Each null genotype was compared to the WT set within an experiment carried out using multivariate linear regression to compare each null genotype to WT, while adjusting for day-to-day differences in treatment conditions. Results were considered to be significant when p ≤ 0.05.

## Results

### Disease Score, Histology and Bacterial Clearance in WT and *Pglyrp*-null Mice

**Fig 1:**
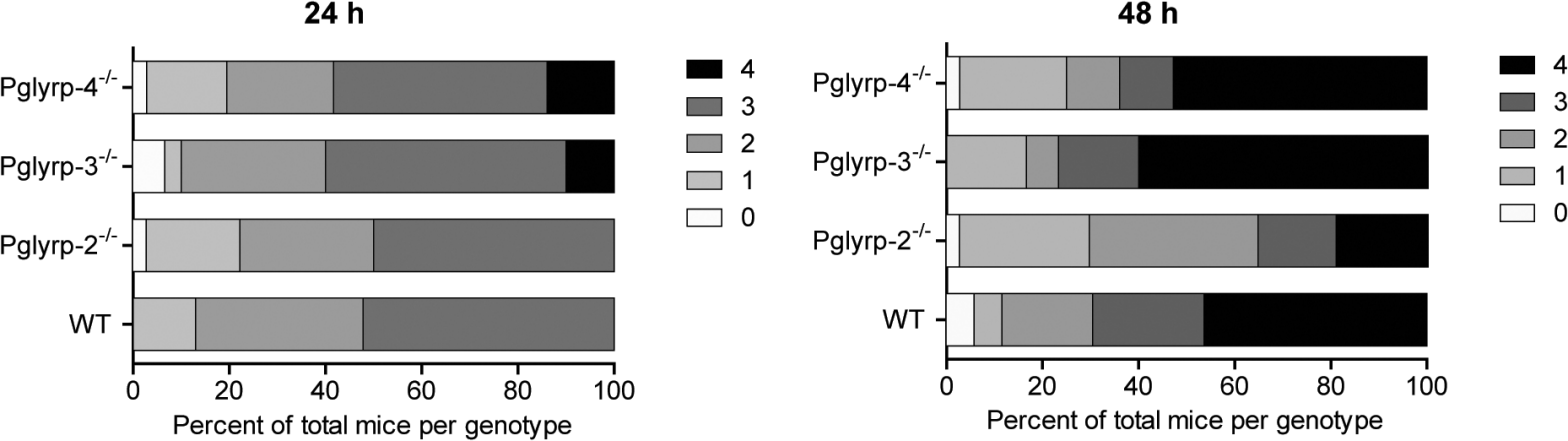
Clinical score frequencies. Clinical disease scores were given in a blinded manner (A) 24 and (B) 48-hours post infection (h p.i.). Results are shown as percent of animals examined in a total of 5 trials per genotype with each trial containing 6–9 animals per genotype. The *Pglyrp*-2^-/-^ mice showed clinical scores comparable to WT in the 24 h.p.i group; the *Pglyrp*-3^-/-^ and -4^-/-^ mice had a few animals with maximal scores by 24 h.p.i suggesting escalated disease. By 48 hours the *Pglyrp*-2^-/-^ mice showed reduced clinical scores.

**Fig 2:**
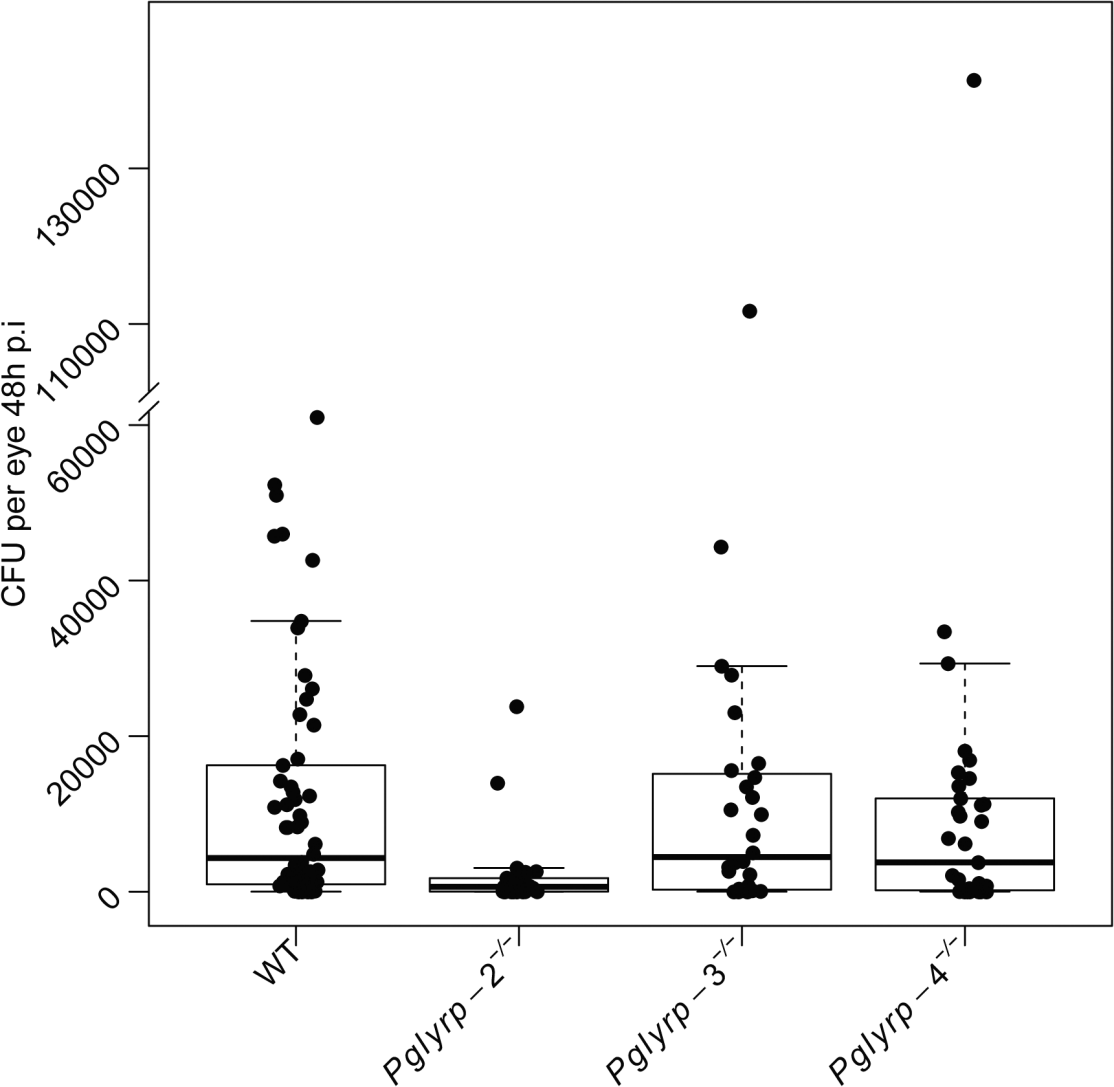
Improved bacterial clearance in the *Pglyrp*-2^-/-^ mice. Dilutions of whole eye homogenates from mice harvested at 48.h.p.i were plated to obtain colony forming unit counts (CFU) per eye. Uninfected eyes did not yield viable bacteria (not shown). The *Pglyrp*-2^-/-^ mouse eyes showed significantly lower viable CFU (t = -3.45, p-value = 0.0007). Bacterial yield in *Pglyrp*-4^-/-^ mice were also lower than WT (t = -1.66, p-value = 0.098). Bacterial yields together with their corresponding clinical scores are shown in S1 Fig.

**Fig 3:**
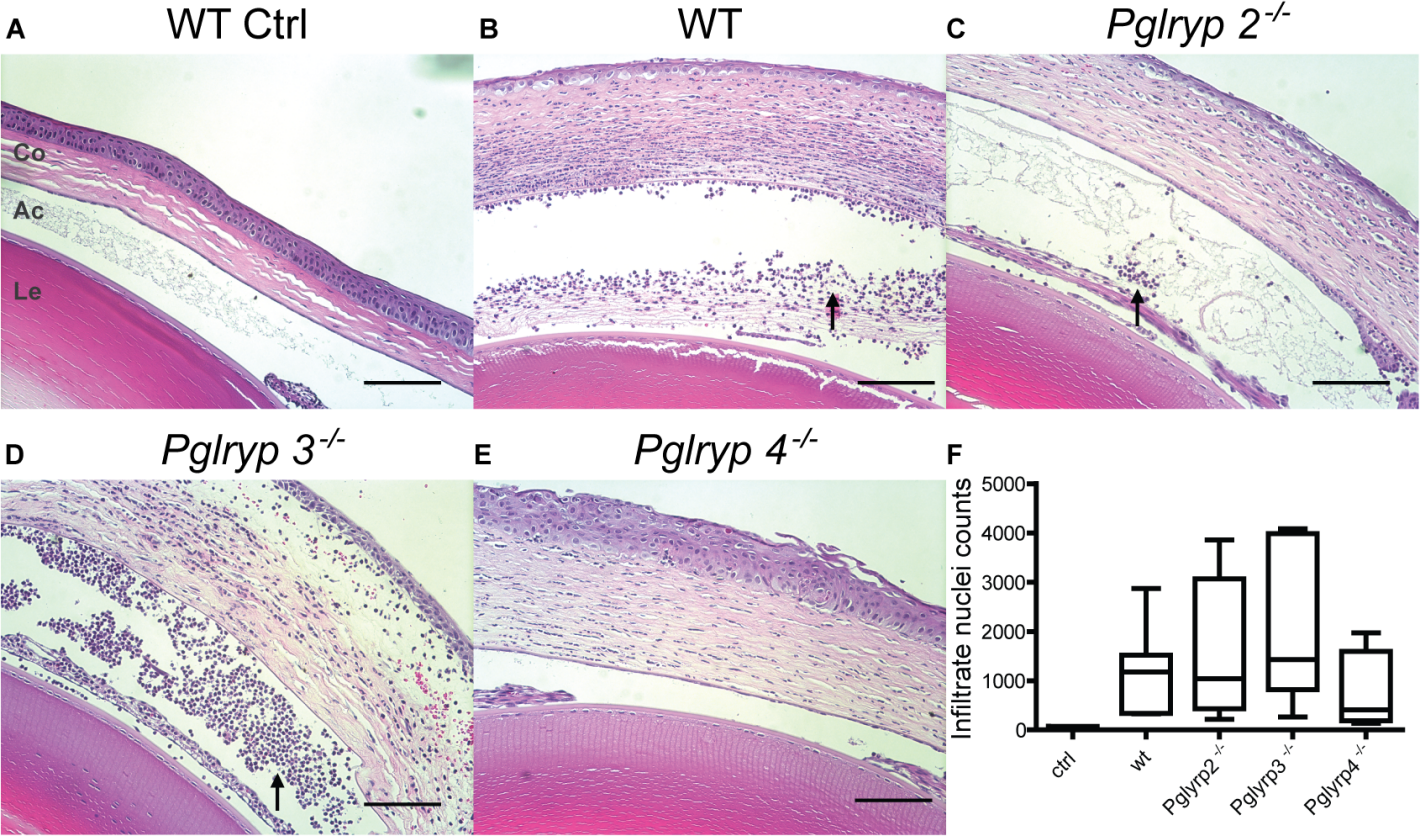
Histology of eyes 48 h.p.i. H and E staining of parasagittal sections through the eye showing the lens (Le), anterior chamber (Ac) and the cornea (Co) (A-E). Cellular inflammatory infiltrates (arrow head) were quantified by counting the nuclei using Image J from 3–5 animals per genotype (F) (additional details provided in S2 Fig).

The eyes were scored in a blinded manner 24 and 48 hours after infections and harvested at the second time point for measurements of viable bacterial yield. Clinical scores after 24 hours of infection were similar in WT and *Pglyrp-2* ^-/-^ mice. However, 10–13% of *Pglyrp*-3^-/-^ and -4^-/-^ mice had higher clinical scores, suggesting that early on disease may progress faster in these two genotypes (Fig 1A). By 48 hours of infection, the *Pglyrp*-2^-/-^ mice showed significantly lower clinical disease scores compared to WT and the other null genotypes (Fig 1B). Viable bacterial yield was similar in the WT and *Pglyrp*-3^-/-^ mice; bacterial yield (Fig 2) was significantly lower (t = -1.66 and p = 0.098) in the *Pglyrp*-4^-/-^ mice, and even more so in the *Pglyrp*-2^-/-^ mice (t = -3.45 and p = 0.0007). We also analyzed viable bacterial yield with their corresponding clinical scores (S1 Fig); in all genotypes animals with a clinical score of 4 had a range of bacterial load while those with a lower clinical score had consistently lower bacteria loads as one would expect. Histology showed robust neutrophil infiltration in infected eyes of all genotypes (Fig 3). We quantified cellular infiltrates in the cornea and the aqueous humor of infected eyes using Image J (S2 Fig) and while there was some variability, overall we found no significant difference between WT and the null mice.

### Pro-inflammatory Cytokines in Infected Eyes

**Fig 4:**
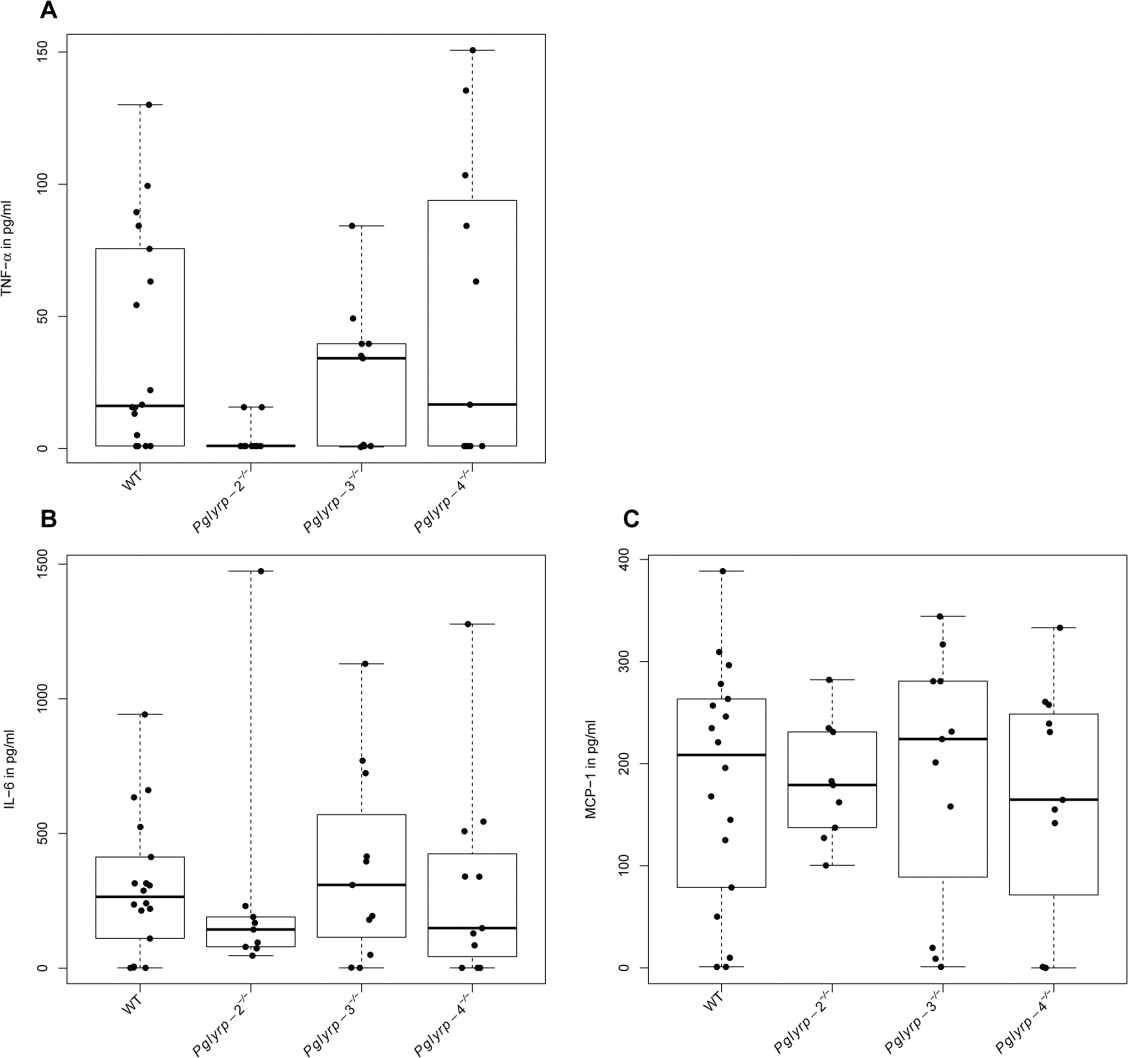
Lower pro-inflammatory cytokine responses in the *Pglyrp*-2^-/-^ infected eyes. Cytokines in whole eye homogenates were measured 48 h. p. i. using a cytometric bead array assay. The results show individual animals from a total of 2–3 trials. Induction of TNF-α was significantly lower (t = -2.4, p = 0.02) in *Pglyrp*-2^-/-^ infected eyes. Median values for MCP-1 and IL-6 were also lower in *Pglyrp*-2^-/-^ mice. Median values for all three cytokines were low in the *Pglyrp*-4^-/-^ infected eyes, but the mean values did not follow this trend.

To determine whether differential cytokine production played a role in the resolution of keratitis in the *Pglyrp*-null mice, we measured a panel of cytokines in whole eye homogenates using a cytometric bead array assay. The cytokines assayed by this kit include IL-12p70, IFN-γ, IL-10, TNF-α, MCP-1 and IL-6. Of these, IL-12p70, IFN-γ and IL-10 could not be detected in infected or un-infected eyes of WT or of the *Pglyrp*-null mouse genotypes. TNF-α, MCP-1 and IL-6 were undetectable in the un-infected eyes (data not shown) and induced in the infected eyes of all genotypes. Induction of TNF-α was significantly lower in infected *Pglyrp*-2^-/-^ eyes compared to WT (t = -2.4, p-value = 0.02), and while IL-6 level was also low, it was not statistically significant due to an outlier. Levels of all three cytokines were also relatively low in the *Pglyrp*-4^-/-^ mice without being statistically significant (Fig 4).

### Differential Expression of *Pglyrp* Genes in Infected Eyes

**Fig 5:**
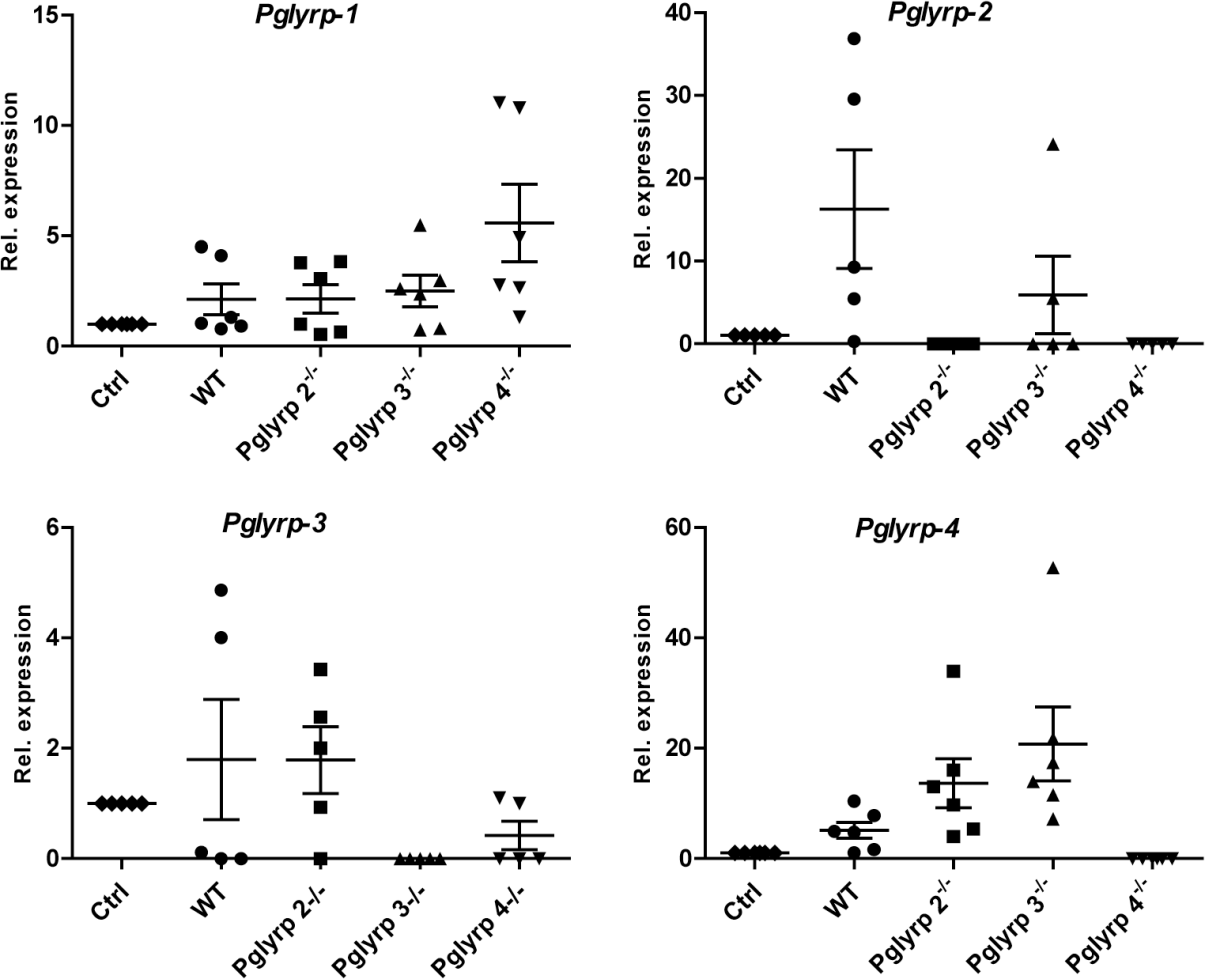
*Pglyrp* gene expressions regulated differentially after infection. Relative expressions of *Pglyrp-1*–*2*, *-3 and -4* compared to *Gapdh* were measured before and 48 h.p.i by qRTPCR on total RNA from six animals per genotype and shown here as mean ± SEM. Fold change was calculated as 2^-ΔΔCt^. *Pglyrp*-1 and -4 expressions were slightly increased after infection but these were not significant.

To determine if expression of any one *Pglyrp* is affected by deficiencies in the other *Pglyrp* genes we used real time qRT PCR on total RNA extracted from whole eyes to measure expression of Pglyrp1-4 in WT and each of the *Pglyrp-*2-4 null mouse eyes before and after infection with *P*. *aeruginosa*. *Pglyrp-1* expression was constitutively high in WT and the *Pglyrp*- 2–4 null mice; after infection its levels increased in all genotypes without reaching statistical significance. *Pglyrp-*2 expression, undetectable before infection, showed variable increases after infection in WT and *Pglyrp* 3^-/-^ mice, and no increase in *Pglyrp* 4^-/-^ mice (Fig 5). *Pglyrp-4* expression was undetectable at baseline and increased variably after infection in WT and *Pglyrp* 2^-/-^ and 3^-/-^ mice.

### Expression of *mBD2*, *mBD3* and *CRAMP*

**Fig 6:**
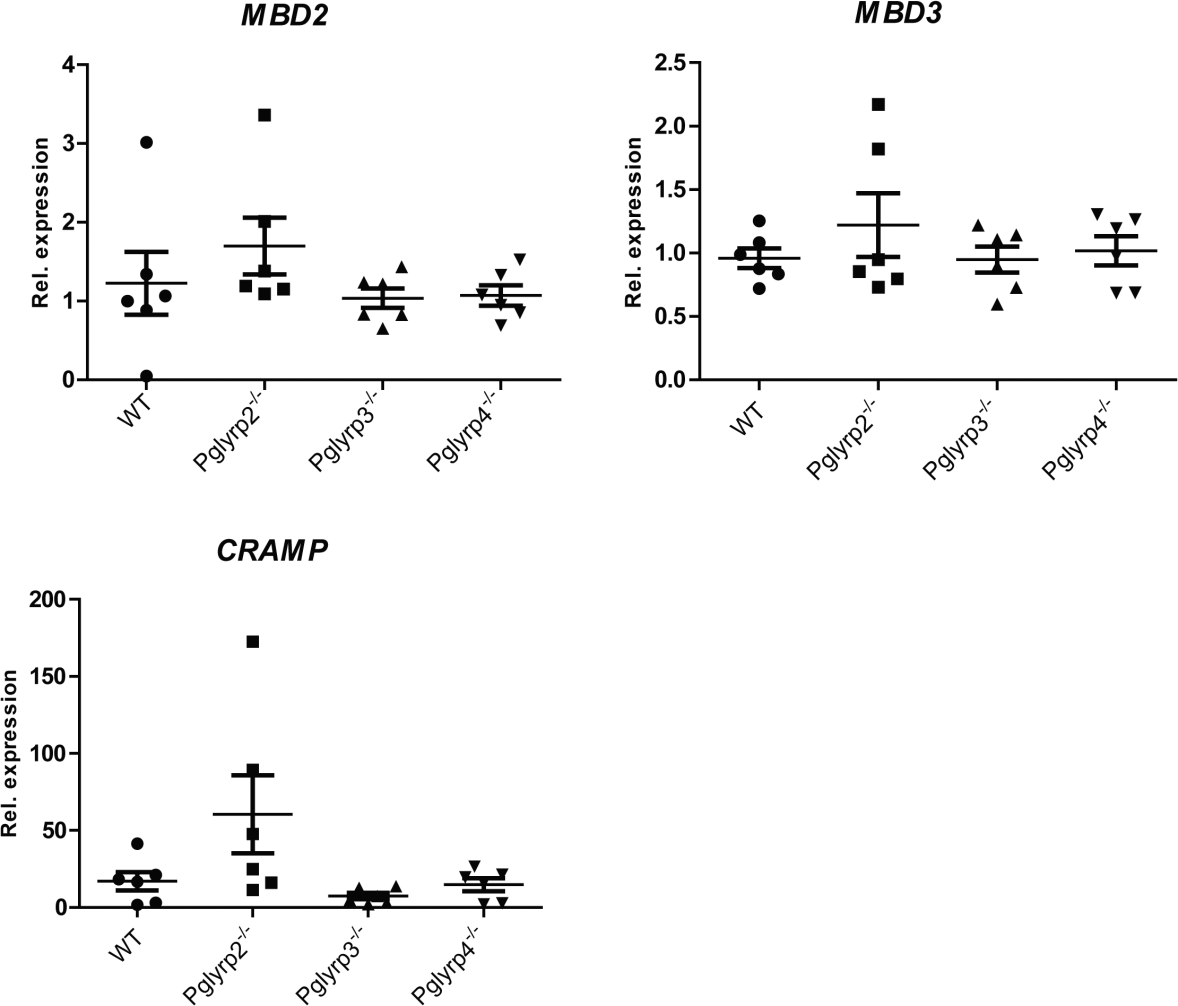
*mBD2*, *mBD3* and *Cnlp* (CRAMP) gene expressions. The results show expression fold change (2^-ΔΔCt^) ± SEM of defensins and *CRAMP* mRNA relative to *Hprt* in total RNA from *Pglyrp-*2 ^-/-^, *Pglyrp-*3 ^-/-^, *Pglyrp-*4 ^-/-^ whole eyes (6 animals per genotype). Expression of *mBD2*, *mBD3* and most notably CRAMP was increased in infected *Pglyrp*-2^-/-^ eyes compared to WT without reaching statistical significance.

We tested if the antimicrobial peptide genes, *mBD2*, *mBD3* encoding β-defensins and *Cnlp* encoding CRAMP (cathelicidin-related antimicrobial peptide) are differentially expressed in the *Pglyrp*- null mice. We performed real time qRTPCR for *mBD2*, *mBD3* and *Cnlp* (CRAMP) before and 48 hours after infection. Compared to WT infected eyes, expressions of all three antimicrobial peptide genes were very low in the *Pglyrp-*3^-/-^ and -4^-/-^ mice (Fig 6). The *Pglyrp-2* ^*-/-*^ mice showed small increases in mBD2 and mBD3 expressions and a pronounced increase in CRAMP, although the data were variable and did not reach statistical significance.

## Discussion

To elucidate the role of Pglyrp proteins in host defense against bacterial infections of the eye, we examined the course of *P*. a*eruginosa* keratitis in mice deficient in *Pglyrp*-2, -3 and -4. Disease clinical scores 24 hours after infection in the WT and *Pglyrp*-2^-/-^ mice were comparably low, whereas 10–13% of the animals from the *Pglyrp*-3^-/-^ and 4^-/-^ groups were scored maximally at 4. This suggests that disease may be somewhat more aggressive in the *Pglyrp*-3^-/-^ and *Pglyrp*-4^-/-^ genotypes, and therefore Pglyrp-3 and -4 may each have a small protective role at the early stages of infection. However, bacterial clearance in the *Pglyrp*-4^-/-^ mice at the later stage was slightly better than WT. The *Pglyrp-*2^-/-^ mice on the other hand showed increased resolution of overall keratitis *and* improved bacterial clearance. Since our previous work elucidated an antibacterial role for Pglyrp-1 [9], we wondered if compensatory over expression of *Pglyrp*-1 could be an underlying factor in the observed improvement in bacterial clearance. *Pglyrp-1* was constitutively expressed in un-infected eyes of WT and in the other null genotypes. Furthermore, after infections *Pglyrp-1* expression increased in the *Pglyrp-*2-4 null and WT mice. In addition, the ocular surface has several antimicrobial peptides, which could also contribute to increased disease resolution in the *Pglyrp*-2^-/-^ mice.

The ocular surface is rich in antimicrobial peptides—the defensins and cathelicidins being the most studied members [15, 16]. These antimicrobial peptides function primarily by bacterial membrane perturbation and can act synergistically with other large antimicrobial proteins [17]. Our findings that transcripts for the antimicrobial peptides mBD2, mBD3 and CRAMP were increased in *Pglyrp-2* ^*-/-*^ may in part explain the observed improvement in bacterial clearance and efficacious resolution of keratitis in these mice. An earlier study on experimental arthritis had noted that *Pglyrp*-2 promoted arthritis by supporting local induction of pro-inflammatory chemokines and cytokines in a TLR4 dependent manner [18]. In our study as well inflammation was low and TNF-α was not induced to the same extent in the *Pglyrp*-2^-/-^ mice as in WT mice. The clinical score is a reflection of inflammation, bacterial count and the resulting corneal clouding. Therefore, the lower clinical disease score in the *Pglyrp-2* ^-/-^ mice could be due to increased bacterial clearance by CRAMP and the defensins expressed at higher levels in the *Pglyrp*-2^-/-^ mice. The exact molecular events underlying the pro-inflammatory role of Pglyrp-2 are not known at the moment, but it likely interacts with innate immune signals mediated by cell surface toll-like receptors (TLRs) and cytoplasmic Nod-like receptors [19, 20].

Taken together, our study suggests that Pglyrp-3 and -4 may have some small protective functions in bacterial keratitis, as mice deficient in these two proteins showed slightly increased progression of keratitis compared to WT mice. *Pglyrp*-2, on the other hand may promote pro-inflammatory cytokines and dampen antimicrobial peptide expression, and the *Pglyrp*-2^-/-^ mice, as a result display a milder form of keratitis. To our knowledge, our study is the first to suggest negative regulations of antimicrobial peptides by Pglyrp-2. The implications of this study are that, *in vivo* expressions of *Pglyrp*-2 must be regulated tightly to balance antimicrobial defense and inflammation to gain a favorable outcome in ocular infections and inflammation.

## Supporting Information

S1 Fig *Pglyrp*-2^-/-^ shows milder keratitis. Clinical score of each animal were plotted on 24 h. p.i (A) and 48.h.p.i (B) together with the viable bacterial yield obtained from the eyes upon harvesting at 48 hours after infection. WT and *Pglyrp*-2^-/-^ mice do not show animals as having maximal clinical scores at the early 24 h. p. i. time point. However, ~ 10–13% of the *Pglyrp*-3^-/-^ and -4^-/-^ mice show maximal clinical scores at this point. By 48. H. p. i. A majority of the WT and *Pglyrp*-3^-/-^ and -4^-/-^ had scores of 3 and 4, whereas several *Pglyrp*-2^-/-^ showed reduced clinical scores by this time point.
(TIF)

S2 Fig Quantification of neutrophil infiltration in ImageJ. A) Raw image, B) Brightness adjusted to reduce collagen and RBC staining. C) Image converted to binary to further reduce non-nuclear signal. D) Anterior chamber to Bowman’s membrane was selected as the area of interest. Particle analysis was applied to the image with 20-pixel minimum, 0.2–1 circularity used as the cut-off. Counted nuclei are highlighted in yellow.
(TIF)
