# Supplementary figures and images for: Functions of Peptidoglycan Recognition Proteins (Pglyrps) at the Ocular Surface: Bacterial Keratitis in Gene-Targeted Mice Deficient in Pglyrp-2, -3 and -4
Source: PLoS One. 2015 Sep 2;10(9):e0137129. doi: 10.1371/journal.pone.0137129 (PMC4558058; doi:10.1371/journal.pone.0137129)

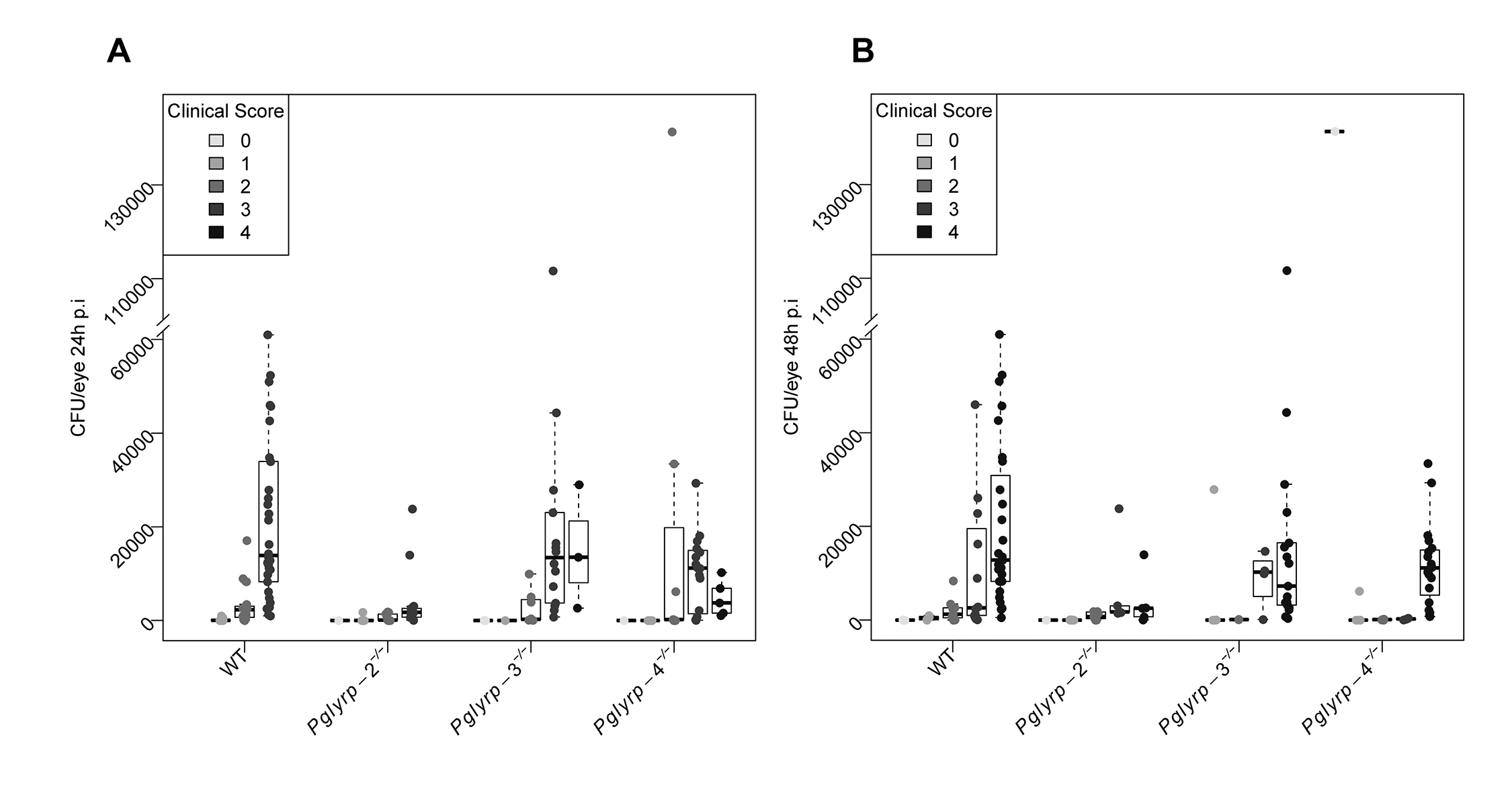

Supplement: S1 Fig — Clinical score of each animal were plotted on 24 h. p.i (A) and 48.h.p.i (B) together with the viable bacterial yield obtained from the eyes upon harvesting at 48 hours after infection. WT and Pglyrp-2-/- mice do not show animals as having maximal clinical scores at the early 24 h. p. i. time point. However, ~ 10–13% of the Pglyrp-3-/- and -4-/- mice show maximal clinical scores at this point. By 48. H. p. i. A majority of the WT and Pglyrp-3-/- and -4-/- had scores of 3 and 4, whereas several Pglyrp-2-/- showed reduced clinical scores by this time point. (TIF) [file pone.0137129.s001.tif]

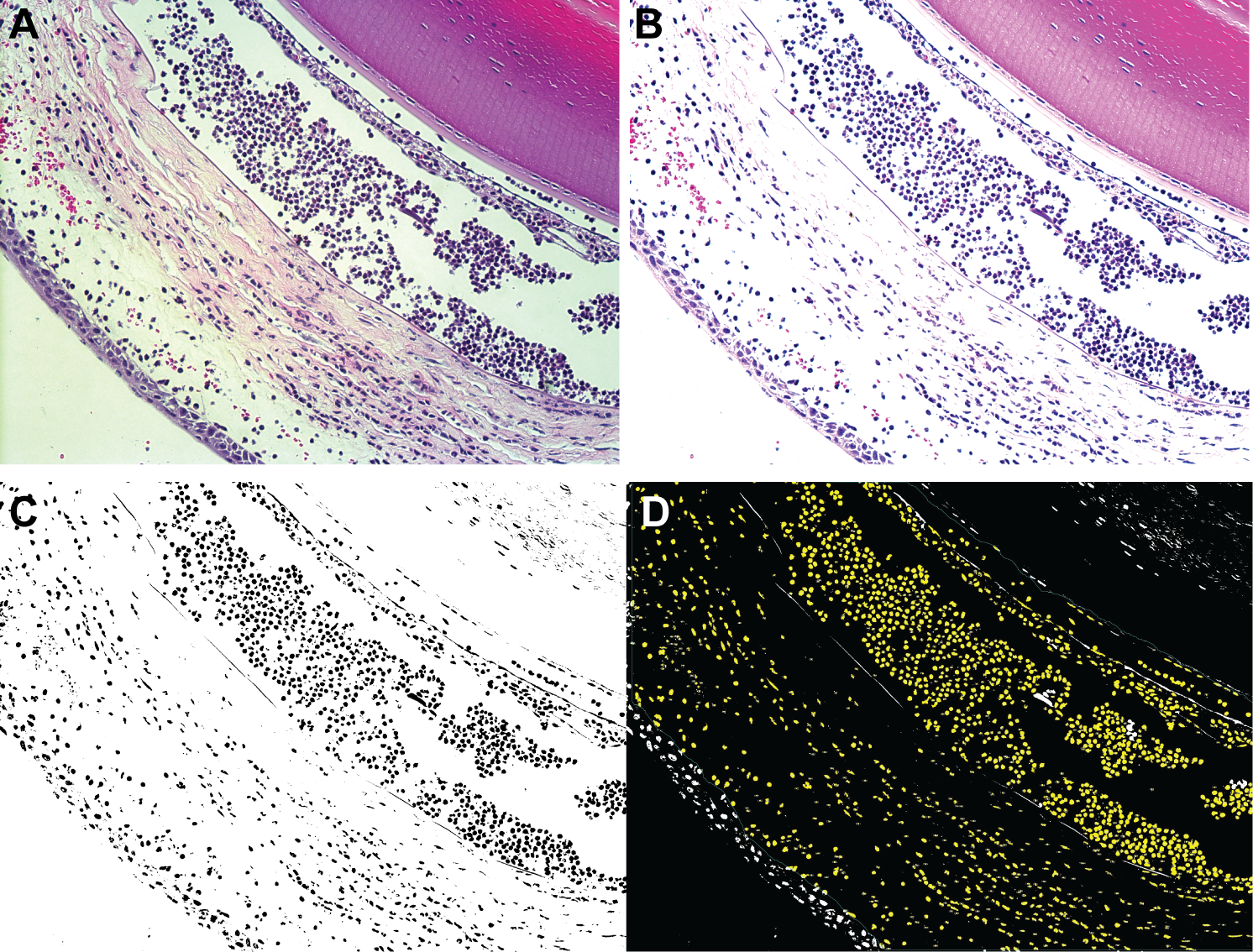

Supplement: S2 Fig — A) Raw image, B) Brightness adjusted to reduce collagen and RBC staining. C) Image converted to binary to further reduce non-nuclear signal. D) Anterior chamber to Bowman’s membrane was selected as the area of interest. Particle analysis was applied to the image with 20-pixel minimum, 0.2–1 circularity used as the cut-off. Counted nuclei are highlighted in yellow. (TIF) [file pone.0137129.s002.tif]
